# Optimizing Research Impact: A Toolkit for Stakeholder‐Driven Prioritization of Systematic Review Topics

**DOI:** 10.1002/cesm.70039

**Published:** 2025-08-14

**Authors:** Dyon Hoekstra, Stefan K. Lhachimi

**Affiliations:** ^1^ Research Group for Evidence‐Based Public Health, Leibniz‐Institute for Prevention Research and Epidemiology (BIPS), Institute for Public Health and Nursing Research (IPP), University of Bremen Bremen Germany; ^2^ Health Sciences Bremen University of Bremen Bremen Germany; ^3^ Department of Special Needs Education and Rehabilitation University of Oldenburg Oldenburg Germany; ^4^ Department of Nursing Management University of Applied Sciences Neubrandenburg Neubrandenburg Germany

**Keywords:** Delphi technique, PICO, priority setting, stakeholder involvement, systematic review, toolkit

## Abstract

**Intro:**

The prioritization of topics for evidence synthesis is crucial for maximizing the relevance and impact of systematic reviews. This article introduces a comprehensive toolkit designed to facilitate a structured, multi‐step framework for engaging a broad spectrum of stakeholders in the prioritization process, ensuring the selection of topics that are both relevant and applicable.

**Methods:**

We detail an open‐source framework comprising 11 coherent steps, segmented into scoping and Delphi stages, to offer a flexible and resource‐efficient approach for stakeholder involvement in research priority setting.

**Results:**

The toolkit provides ready‐to‐use tools for the development, application, and analysis of the framework, including templates for online surveys developed with free open‐source software, ensuring ease of replication and adaptation in various research fields. The framework supports the transparent and systematic development and assessment of systematic review topics, with a particular focus on stakeholder‐refined assessment criteria.

**Conclusion:**

Our toolkit enhances the transparency and ease of the priority‐setting process. Targeted primarily at organizations and research groups seeking to allocate resources for future research based on stakeholder needs, this toolkit stands as a valuable resource for informed decision‐making in research prioritization.

## 1 Introduction

A Cochrane key principle guiding the production of systematic reviews is “striving for relevance”. This principle emphasizes the importance of producing reviews that are not only methodologically sound but also directly relevant and applicable to real‐world contexts [1, 2]. Hence, for Cochrane reviews the inclusion of a broad set of stakeholders is essential in the production of systematic reviews and in selecting which review topics are relevant [3, 4, 5].

To search and select the most relevant research topics from a more extensive list of proposed options, research priority setting (RPS) exercises are conducted [6, 7, 8, 9]. A number of approaches and frameworks for RPS exist varying by aim, target group and level of the RPS exercise [6, 9, 10, 11, 12]. A structured approach for RPS is essential to prevent arbitrariness and subjectivity in the selection process to obtain a robust outcome [11] and can serve as a platform that facilitates interaction and trust‐building among diverse stakeholders, which are critical for the successful integration of research into decision‐making processes [13, 14, 15].

Although research groups or institutions who are conducting or funding systematic reviews would greatly benefit from structurally prioritizing topics, structured frameworks that focus specifically on setting priorities for systematic reviews are rare [3, 14, 16]. Fadlallah and colleagues examined approaches used for prioritizing topics for systematic reviews and other types of evidence syntheses. They highlighted seven studies that proposed a structured RPS approach, include steps such as defining the purpose and scope of the RPS, reviewing the literature, and including stakeholder input. One of the examined approaches for prioritizing topics for systematic reviews developed a generalized tool, in which a pre‐selected pool of questions for reviews are prioritized preferably in a group setting where stakeholders are physically together [14].

Also, within Cochrane, review groups use different approaches and methods to evaluate which review topics are relevant and need to be prioritized [2, 3, 17]. Nevertheless, research on this topic revealed that 23 out of 52 Cochrane review groups surveyed had no prioritization process in place for review topics at all, and only 13 out of 52 utilized a transparent and structured process for prioritizing their review work [3]. The approaches used by these 13 review groups varied from identifying priority topics based on health status data, derived from recommendations of clinical guidelines or other systematic reviews, or extracted from evaluating gaps in existing evidence coverage. However, most structured approaches focused on soliciting suggestions on priorities from stakeholders, e.g. review groups, clinicians, consumers and policy makers [3].

Even though the emphases of these different approaches are highly relevant to the prioritization of review topics, they limit the scope of an RPS if they are applied individually. Therefore, these approaches ought to be combined in one RPS approach, either as separate stages or as assessment criteria [18, 19].

One of the challenges when prioritizing topics for systematic reviews is the initial difficulty for many stakeholders to phrase systematic review questions with enough precision to be feasible, in particular for stakeholders with little or no practical experience in developing systematic review topics.

Therefore, we aimed to develop a pragmatic framework to develop and prioritize topics for systematic reviews in a way that is 1) user‐friendly for researchers and review teams who want to conduct such a priority setting exercise, 2) that can be implemented on a larger scale to limit time and costs involved and 3) that allows for the inclusion of a wide variety of stakeholders with varying expertise in working with systematic reviews. To do this, we developed an approach that helps to structure the proposed topics in the RPS according to the PICO framework (PICO stands for Population or Problem; Intervention, Item of Interest, Exposure, or Issue; Comparator; Outcome).

This paper introduces a toolkit for the pragmatic development and prioritization of topics for systematic reviews incorporating a wide range of stakeholders, including the preparatory stage for the RPS exercise and all the complementary material required to transfer and implement the RPS into a different field of interest. It incorporates the key fundamental aspects of a structured RPS and applies them to the specific challenges of prioritizing systematic review topics.

The approach presented in our framework has been developed and applied in collaboration with Cochrane Public Health Europe (CPHE), a subdivision of Cochrane Public Health [18, 19].

**Figure 1:**
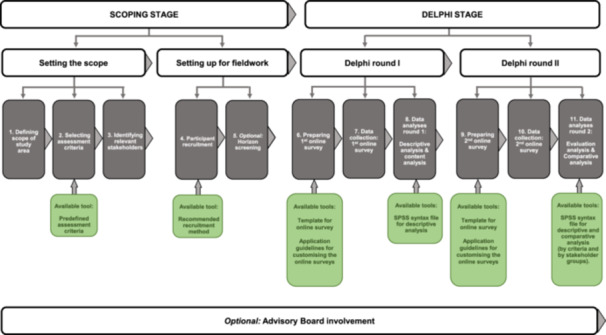
Visualization of the scoping and Delphi stage.

## 2 Material and Methods

Our structured framework consists of multiple steps for prioritizing systematic review topics and multiple tools that support conducting the RPS exercise in a different setting. Overall, our framework encompasses first two scoping rounds and then two Delphi rounds (See Figure 1) (for general methodological guidance on the Delphi technique see [20, 21, 22]). The different steps and their application are outlined in a comprehensive and detailed step‐by‐step guide below.

1. Optional: Establishing an advisory board
  We propose to start by establishing an advisory board to guide the research process. It is crucial that this group consists of both technical and methodological experts [4, 8, 23]. The advisory board can provide added quality to the process by ensuring the relevance and feasibility of the RPS exercise, contributing to the scoping of the RPS (steps 1 to 5 below), and fulfils an essential task by potentially aiding the analysis and interpretation of the research team after both Delphi rounds (step 8 and 11 below) [8, 10, 23, 24].
2. Defining the scope of the research area
  First, set the scope of the field of interest for the RPS exercise [25, 26]. The research team and potentially the advisory board have to identify exactly in which specific research area they intend to prioritize the review topics. It is helpful to split the research area conceptually in two or more domains, each representing different dimensions of the research area. Outlining (multiple) domains encourages participants to think of topics along all dimensions that could be considered within the given research area, including dimensions they would not have considered themselves directly when asked ad hoc to propose topics. The set scope frames the further process of the RPS exercise.
3. Selecting assessment criteria for prioritizing systematic review topics
  The next step is to specify assessment criteria that will be included in the rating of review topics. The inclusion of assessment criteria in the RPS is an essential element of our framework. Rating of topics with assessment criteria effectively facilitates the prioritization process [8, 16, 27]. However, a single rating criterion to gauge the importance of a topic is usually too simplistic. By defining multiple assessment criteria different aspects of a topic can be rated [27, 28]. The choice of assessment criteria depends on the field of the RPS [29, 30]. An example of which assessment criteria can be applied in a certain research area can be found in Box 1.
4. Identifying relevant stakeholders
  By engaging multiple stakeholder groups, the prioritization process benefits from a broader and more comprehensive perspective. Relevant stakeholder groups may encompass (representatives of) researchers, clinicians, policymakers, consumers, patients, and other entities with a vested interest in the subject matter under consideration [13, 31].
5. Participant recruitment ‐ Recommendation
  We propose to recruit participants by approaching organizations within each stakeholder group. Once the relevant organizations for each stakeholder group have been identified, recruitment of participants can start. Each included organization is requested to nominate one or more participants from within their organization for the Delphi stage [18, 32, 33]. We suggest to restrict the number of nominated participants to three per organization to avoid potential bias (for larger organizations it is easier to nominated a larger number of participant than smaller organizations).
  Before the start of the Delphi stage, the nominees themselves have to be informed about the objective of the research and their voluntary and anonymous participation. The surveys in the Delphi stage can only be completed after their notified consent has been obtained.
6. Optional: Horizon screening
  As an optional and additional extension to the scoping stage, we recommend horizon screening: Collecting statements and existing recommendations from existing literature about topics for future systematic reviews within the set scope. We suggest to follow the approach used by [4]: Screening explicit electronic databases and sources containing high quality systematic reviews that fit the scope of the RPS exercise. During this process the recommendations in the identified reviews are analyzed for statements on gaps in the existing literature. These gaps can then be categorized into the domains that have been defined in step 1. These identified review topics can either serve as reference point for the participants in the first Delphi round or can be used in the first Delphi round as a list of topics that can be selected for prioritization by the participants.
7. Preparing online survey Delphi round 1
  After the decisions have been made in the scoping stage (steps 1 to 5 above) they need to be integrated in the online survey of the first Delphi round: The descriptions of the field of study and possible domains within the field (step 1), the predefined assessment criteria (step 2), and the (possibly) pre‐selected topics from the horizon screening (step 5), need to be inserted in the text of the online survey (see Supporting file 1 for the template of the first round survey). Furthermore, the contact details of the recruited participants (step 4) can be inserted in the online survey software to automatically send anonymized invitations to the online survey.
8. Data collection Delphi round 1
  Once the online survey is programmed and participants have been invited, the survey of the first Delphi round will be available online. We advise to have the first online survey online and operable for up to 4 weeks and send email reminders approximately every 7 days after the first invitation.
  To identify review topics that in the participants' opinion should be considered as potential priority topics, they are encouraged to propose topics for systematic reviews within the set scope themselves and optionally select topics from the preselected list of topics generated by the horizon screening (step 5). This is repeated for every defined domain within the field of interest (step 1). Because the domains serve as guidance for participants to consider the diverse realm of the set scope of the RPS exercise, the order of the presented domains should be randomized.
  Given that some stakeholders may lack familiarity with systematic reviews, our toolkit provides them with structured guidance to navigate the process. Specifically, we created a template corresponding to each component of the PICO format for formulating systematic review topics.
  To facilitate comprehension and application, we accompanied each PICO component with an explanation and an illustrative example, thereby assisting participants in a stepwise fashion as they determined the components of their proposed topics.
  Additionally, the participants are asked to identify assessment criteria: They can select from the predefined assessment criteria in step 2 or propose further criteria that they deem necessary for assessing the importance of review topics within the set scope.
9. Data analyses Delphi round 1
  The results derived from Delphi round 1 will encompass an expanded inventory of review topics: either chosen or put forth by the stakeholders. A content analysis of the proposed topics and PICO components is necessary to first reduce the overlap between the topics and cluster them within the domains identified in step 1. Subsequently, the proposed topics and proposed PICO components need to be transformed into specific topics for systematic reviews [34, 35].
  Additionally, a descriptive analysis can be used to investigate the participants' characteristics, such as profession, type of function, experience in the field of interest, and experience with and knowledge about systematic reviews. Furthermore, the review topics derived from the content analysis can be counted, to see which topics and domains are most selected and proposed.
  Also, the selected and proposed assessment criteria need to be aggregated into distinct assessment criteria before they are counted to decide which criteria are deemed most useful for assessing review topics in Delphi round 2.
10. Preparing online survey for Delphi round 2
  The results from the data analyses in step 8 should be integrated in the online survey of the second Delphi round (see Supporting file 2 for the template). All individuals nominated for participation in Delphi round 1 are invited to join the second Delphi round, regardless of their participation status in the first round.
11. Data collection Delphi round 2
  In Delphi round 2, participants evaluate the review topics based on the stakeholder‐refined assessment criteria on a scale ranging from 1, signifying “totally agree,” to 4, indicating “totally disagree”. Alternatively, they can choose the option “I cannot assess this”.
  Additionally, participants are requested to assign a weight to the importance of the criteria employed for evaluating review topics, using a 100‐point scale. The weight indicates the relative importance of each assessment criterion and can highlight potential differences or similarities between participants individually or, when averaged out by stakeholder group, potential differences or similarities between stakeholder groups [18, 27].
12. Data analyses Delphi round 2
  For each topic, the average rating by assessment criterion can be calculated. An example of the rating comparison by assessment criterion applied in actual RPS exercise can be found in Box 2. However, additional analysis can be done. For example, the average topic rating and also the average weighting for each assessment criterion can be computed by stakeholder group to highlight differences between stakeholder groups [18, 32]. Also, the participants' characteristics, such as profession, type of function, experience in the field of interest, and if the person participated in the first Delphi round, may be used to identify patterns in the rating score. For the intra‐stakeholder group comparison, to observe if there is consensus within each stakeholder group itself, several statistical procedures exist [36, 37].

Box 1 **Example for scoping stage—based on the application of the RPS study in Switzerland [18]**

- Research area:
  − **Public health**

- Domains (based on the classification of the European SPHERE project:
  − **Prevention** (surveillance, modelling, disease control)
  − **Health promotion** (information, skills, behaviour, environment),
  − **Health services** (medical care, systems, services).

- Assessment criteria:
  − **Improving the health of the population** → Systematic reviews regarding the topic can lead to measures that contribute to improving the health of the population (e.g., reducing the burden of disease, promoting physical and mental health).
  − **Health equity** → Systematic reviews regarding the topic can lead to measures that contribute to more health equity.
  − **Insufficient research to date** → Systematic reviews regarding the topic may indicate that research results on this topic are insufficient (e.g., are not available, are of insufficient quality, are not up to date or will become more important in the future).
  − **Effect on public health if successful** → Systematic reviews regarding the topic can have a positive effect on further research or practice in public health.
  − **Potential for innovative action** → Systematic reviews regarding the topic have a high potential for innovative findings

- Stakeholder groups:
  − **Research and/or higher education**
  − **Administration and/or politics**
  − **Health organisations representing certain target groups in the population**
  − **Organisations representing healthcare professionals and institutions**
  − **Health insurers**

Box 2 **Example of the results (criteria comparison) from applying our RPS framework [18]: Ranking differences between criteria for the top 15 review topics (Public health, Switzerland).**

**Table:** 

Review topic (total number of times assessed)	Overall		A. Improving the health of the population		B. Health equity		C. Insufficient research to date		D. Effect on public health if successful		E. Potential for innovative action	
	R	M	R	M	R	M	R	M	R	M	R	M
Improved access to prevention services for specific target groups (159)	**1**	**1.47**	3	1.41	2	1.21	5	1.48	48	1.88	2	1.37
Cognitive training against dementia diseases (156)	**2**	**1.63**	8	1.48	26	1.59	1	1.32	85	2.11	15	1.63
Highlight target‐group‐specific good practices in vulnerable groups (163)	**3**	**1.64**	37	1.73	1	1.19	87	2.06	12	1.50	20	1.72
Better education for relatives of people with a mental illness (166)	**4**	**1.65**	11	1.55	7	1.38	9	1.57	98	2.19	18	1.67
Mental health education for adults (165)	**5**	**1.66**	15	1.59	118	2.05	4	1.47	16	1.53	10	1.59
More education about e‐cigarettes/oral tobacco (SNUS) for children and adolescents (156)	**6**	**1.67**	1	1.34	4	1.30	44	1.84	58	1.97	37	1.88
Peer‐to‐peer education on health risks from drugs (171)	**7**	**1.69**	96	1.91	58	1.77	46	1.84	17	1.56	4	1.44
Integration with equal opportunities of chronically ill people into society (157)	**8**	**1.71**	57	1.81	60	1.79	20	1.70	2	1.36	33	1.87
Information in schools about the correct use of mobile phones (167)	**9**	**1.72**	18	1.62	57	1.77	49	1.86	28	1.69	17	1.66
Improving health literacy of nursing home staff in taking care of elderly people with mental disorders (158)	**10**	**1.72**	9	1.52	86	1.88	74	2.00	19	1.57	7	1.55
Provide healthy food in schools and workplaces (170)	**11**	**1.75**	21	1.63	62	1.79	57	1.91	38	1.79	16	1.65
Improved education about sexual health for specific target groups (162)	**12**	**1.75**	6	1.45	10	1.39	21	1.70	138	2.38	39	1.90
Better access to adequate care for marginalized groups (169)	**13**	**1.76**	17	1.62	15	1.46	31	1.74	150	2.44	11	1.59
Increased early recognition structures of mental illnesses (136)	**14**	**1.78**	14	1.59	120	2.06	52	1.88	11	1.50	13	1.61
Mental health resilience training (161)	**15**	**1.79**	10	1.54	67	1.81	3	1.46	149	2.43	25	1.79
Better access to good quality clinics for vulnerable groups in poor countries (158)			113	1.97	49	1.73	201	2.56	1	1.32	234	3.07
Support for group practices with interprofessional cooperation (163)			5	1.44	114	2.03	43	1.84	207	2.72	1	1.35
**Mean (of all 245 review topics):**		**2.21**		**2.12**		**2.13**		**2.22**		**2.27**		**2.33**

M = Mean; R = Rank

The bold values in the first two columns stipulate the overall rank and mean of the top‐15 rated topics for all criteria combined. The values in the following columns present the rank and mean of these topics by criteria. The final row highlights the mean of the rating of all 245 topics.

## 3 Results

We have compiled a comprehensive set of ready‐to‐use tools to facilitate both the creation of the survey and the subsequent data analyses. These tools are designed to facilitate the efficiency and accuracy of the research efforts. Besides a list of predefined assessment criteria (see Box 1 and Supporting file 1) and a recommended recruitment method to achieve high participation rates, we provided the following tools:

- A template for the online surveys as a Microsoft Word file (Supporting files 1 & 2). This serves as a basic document in which all questions, response options and the survey flow are clearly presented.
- In addition to the Word document, we have also provided the survey questionnaire structure file (.lss) (Supporting files 3 & 4). This format is easy to import and export, making it straightforward to integrate your survey into different platforms.
- To understand correctly how to apply our surveys, we established a modification guideline (Supporting file 5). This ensures that one can adjust the templates according to their specific scope of their RPS exercise and that any required adjustments to the surveys are made accurately and consistently.
- To comprehensively evaluate the data, we have developed a syntax for the descriptive and comparative analyses in SPSS software (Supporting file 6). These analyses cover a range of perspectives, including overall evaluation of the assessments, evaluation according to specific criteria, and evaluation according to different stakeholder groups. This multidimensional approach enables a comprehensive evaluation of the survey results.

## 4 Discussion

A few approaches and frameworks for prioritizing topics for evidence synthesis exist, including approaches that gather priority topics by reviewing literature, by soliciting suggestions on priorities from stakeholders, or by rating potential topics from an existing pool of topics [3, 14, 16, 17]. The emphases of these different approaches are in itself useful in RPS for systematic reviews, however, we combined existing best practices into a single RPS framework that also allows for the participation of a large panel of relevant stakeholders in easy‐to‐conduct and easy‐to‐replicate online surveys [18, 19].

Overall, our framework allows for a feasible, yet rigorous insight into the differences in prioritization preferences of various stakeholders and provides valuable information on the underlying reasons for these differences by using multiple assessment criteria, as recommended by previous studies [8, 16, 27, 28]. Each stakeholder group may manifest unique priorities and conducting a comparative analysis allows researchers to gain valuable insights into the varied perspectives and priorities associated with the prioritized review topics.

One of the main objectives of this RPS framework is to promote the involvement of a broad range of stakeholders in the prioritization process. To also ensure the inclusion of individuals who might be less familiar with systematic reviews, we recommended several strategies:

The first one is the integration of a novice recruitment strategy as successfully applied by [18, 32, 33]. In this recruitment method, contact persons within the identified organizations are asked to nominate participants for the Delphi stage. We communicate in our invitation that those being nominated should have the appropriate expertise related to the research area—not necessarily expertise in systematic reviews—to participate in the study and provide insightful responses to the research subject. This strategy increases the likelihood that the invited individuals will participate and it allows for a sampling of individuals thought to possess the most expertise to contribute to the RPS exercise.

Second, our framework aims to ensure a low threshold for involvement. As it can be assumed that some stakeholders might not be familiar with the PICO format for phrasing systematic review questions, the stakeholders are guided step‐by‐step to indirectly create potential review topics in a PICO format. In a study that implemented this framework, the high participation rates (out of 215 individuals that were nominated, 91.6% participated in the first Delphi round and 77.7% in the second Delphi round) and the large number of suggested topics for systematic reviews (*n* = 728) demonstrates that with this approach it is feasible to involve many stakeholders in developing and prioritizing review topics. Several respondents also highlighted the importance of such an RPS exercise and expressed their gratitude to be able to participate in the feedback at the end of the RPS exercise [18].

To enable the inclusion of many individuals, a modified Delphi technique is applied to prioritize topics for systematic reviews. Our modified Delphi procedure is a reductionist form of the usual Delphi study. A respondent can consider other participants suggestions or judgement when making the final assessment in the second online survey. In principle a third round would be possible, but we advise against it to keep the workload for the participants at an acceptable balance. In the strictest sense the result of our approach is not a (Delphi) consensus, but reveal stakeholder preferences that were also confronted with other stakeholder holder preferences while making judgements. Nevertheless, our method increases the inclusion of (a large number of) stakeholders who would otherwise have potentially been left out of the discussion in a full Delphi study. Also, possible top experts for a Delphi study could fall through the cracks in the non‐modified process [38, 39].

The toolkit can be implemented in several research areas as the main principles remain the same across disciplines. Given the possible heterogeneity of research areas and stakeholder preferences, the toolkit offers the flexibility of either using predefined assessment criteria only or creating custom criteria targeted for specific research areas.

Approaching pre‐identified stakeholder groups and having anonymous respondents does not give a representative sample as with a truly random sampling; something which is very difficult to do when surveying subject matter experts as the true population of all subject matter experts is unknown. Nevertheless, the investigators can improve the representativeness of the RPS exercise by increasing the number of the stakeholder groups that are invited. Our online approach has—in principle—no limit in the number of participants and is very easily scalable.

Finally, it is important to note that we were not able to assess if and how outcomes from applying the proposed RPS framework affects the actual systematic reviews that are conducted within the set research area. In general, a key limitation of patient and public engagement in RPS is the lack of evaluation data on the success and extent in which stakeholders are involved [15]. Future research should investigate how participants assess finished systematic reviews to see whether it meets their expectations and priorities to empirically validate the usefulness of our framework. Additionally, an evaluation of the applied assessment criteria can support the development of a set of validated assessment criteria for individual research areas.

## 5 Conclusion

This paper presents a pragmatic toolkit designed to streamline the prioritization of topics for systematic reviews across various research fields, including health, education, and social welfare. By integrating a structured, multi‐step framework that employs a modified Delphi technique, our approach facilitates the inclusion of a broad range of stakeholders, ensuring that the prioritization process is both collaborative and transparent.

Key to our framework is its adaptability and scalability, allowing for customization to fit specific research contexts while minimizing resource demands. The toolkit's compatibility with freely available survey software further enhances its accessibility and ease of implementation.

The adaptability, ease of use, and focus on inclusivity make this toolkit a valuable resource for organizations and research groups committed to conducting relevant and impactful systematic reviews informed by the needs of their respective fields.

## Author Contributions

**Dyon Hoekstra:** conceptualization, data curation, formal analysis, investigation, methodology, project administration, software, visualization, writing – original draft, writing – review and editing. **Stefan K Lhachimi:** conceptualization, data curation, methodology, supervision, writing – review and editing.

## Conflicts of Interest

(See ICMJE COI disclosure forms & CESM Disclosure of Interest form). The other authors declare no conflicts of interest.

## 1 Peer Review

The peer review history for this article is available at https://www.webofscience.com/api/gateway/wos/peer-review/10.1002/cesm.70039.

## Supporting information


**Supplementary file 1:** Template online survey round 1.

**Supplementary file 2:** Template online survey round 2.

**Supplementary file 3:** Template Prioritising for SR Round 1.

**Supplementary file 4:** Template Prioritising for SR Round 2.

**Supplementary file 5:** Modification guideline Online surveys priority setting for systematic review topics.

**Supplementary file 6:** Syntax for analyses Toolkit RPS.

## Data Availability

The data that support the findings of this study are available from the corresponding author upon reasonable request.
