# Supplementary material for: Optimizing Research Impact: A Toolkit for Stakeholder‐Driven Prioritization of Systematic Review Topics
Source: Cochrane Evid Synth Methods. 2025 Aug 14;3(5):e70039. doi: 10.1002/cesm.70039 (PMC12362723; doi:10.1002/cesm.70039)
Supplement: Supplementary file 1 — Template online survey round 1. [file CESM-3-e70039-s005.pdf]

"Prioritising topics for systematic reviews"
Template for online survey round 1

(Fill in names and affiliations of the research team)

Section A: Introduction

Welcome to the first survey on: “Prioritising topics for systematic review”

Please read the following information before you agree on participating in this survey!

Thank you for your participation in our first round of our Delphi study on prioritising topics for systematic reviews in the field … (*fill in field of study*).

We, ... *(fill in inviting institutes)*, hereby cordially invite you to share your expertise as an important stakeholder with regard to the research needs in … (*fill in field of study*). We would like you to tell us which review topics should be prioritised. The survey will take about 15 to 20 minutes.

This survey is conducted …*(fill in goal of the study)*.

In order to avoid multiple answers from individual participants, you can only fill out the survey once. However, on each of the following pages you can select the "continue later" option at the top of the website to save your answers to continue later. Your answers are saved for you even if you switch off your computer.

All data will be … *(fill in information regarding data protection and privacy)*.

If you have any questions or concerns, please do not hesitate to contact:

… *(fill in contact details)*

If you agree to this and would like to participate, then please click on “next”.

Section B: General information

<u>Question 1 - Which position do you hold within your organisation?</u>
Please choose the answer that best describes your situation in your main profession.

- *Employee*
- *Project leader*
- *Working group or department head*
- *Head of the organisation*
- *Other (please specify):*
- *No answer*

<u>Question 2 – Which of the following options would you consider most appropriate for describing your profession?</u>
Please choose the answer that best describes your situation in your main profession.

- *Research and/or higher education*
- *Governmental bodies and/or politics*
- *… (fill in field of study) related NGO & NPO and/or representatives of the public (for example: patient associations)*
- *Representative of … (fill in field of study) professionals*
- *… (fill in field of study) insurance*
- *Other (please specify):*
- *No answer*

<u>Question 3 – At what level do you fulfil the majority of your activities?</u>
Multiple answers possible

- *Local / Regional level*
- *National level*
- *International level*
- *No answer*

<u>Question 4 – How many years have you been working in the field of …</u> *<u>(fill in field of study)</u>***<u>?</u>**
If you do not consider yourself as active in this field of study, then please fill in "0"

Section C: Collection of topics to be prioritised (per domain)

Question 5

In the following section, we will ask you which topics you believe should be considered as potential priority topics for systematic reviews in the field of … *(fill in field of study)*.

Potential topics for this survey are divided into different domains. This division is based on a previously conducted literature study and on the expert opinion of a scientific advisory board. The domains are listed below:

1. …
2. …
3. …

Etc.

We will ask you to suggest topics that you believe should be prioritised within these domains on the following pages.

<u>Domain 1:</u> … *(fill in name of domain)*
Our definition of this domain:

This domain includes … (fill in description of the domain)

Key terms in this domain:

- *… (fill in key terms and/or examples for this domain)*
- …
- …

<u>Which potential topics within the domain "</u>*… (fill in name of domain 1)***<u>" should be considered as a high priority? Please do not hesitate to propose multiple topics.</u>**
Please fill in only one topic per row
Multiple answers possible – Maximum 5 topics

Topic 1:

What is your particular interest in this topic?

What type of intervention / phenomena?

For whom? Who is the target group?

How should the success of the intervention be measured? Which factor needs to be assessed concerning this phenomenon?

(Similar sub questions for topic two, three, four, and five)

<u>Domain 2:</u> … *(fill in name of domain)*
Our definition of this domain:

This domain includes … (fill in description of the domain)

Key terms in this domain:

- *... (fill in key terms and/or examples for this domain)*
- …
- …

<u>Which potential topics within the domain "</u>*… (fill in name of domain 2)***<u>" should be considered as a high priority? Please do not hesitate to propose multiple topics.</u>**
Please fill in only one topic per row
Multiple answers possible – Maximum 5 topics

Topic 1:

What is your particular interest in this topic?

What type of intervention / phenomena?

For whom? Who is the target group?

How should the success of the intervention be measured? Which factor needs to be assessed concerning this phenomenon?

(Similar sub questions for topic two, three, four, and five)

Domain 3: … *(fill in name of domain)*
Our definition of this domain:

This domain includes … (fill in description of the domain)

Key terms in this domain:

- *… (fill in key terms and/or examples for this domain)*
- …
- …

Which potential topics within the domain "*… (fill in name of domain 3)***" should be considered as a high priority? Please do not hesitate to propose multiple topics.**
Please fill in only one topic per row
Multiple answers possible – Maximum 5 topics

Topic 1:

What is your particular interest in this topic?

What type of intervention / phenomena?

For whom? Who is the target group?

How should the success of the intervention be measured? Which factor needs to be assessed concerning this phenomenon?

(Similar sub questions for topic two, three, four, and five)

(Add similar blocks as above in case more domains are defined)

Section D: Adapting domains

Question 6 – We have asked you to propose topics within the three pre-set domains "… *(fill in name of domain 1)*", "… *(fill in name of domain 2)*" and "… *(fill in name of domain 3)***". Did you miss certain domains in the** … *(fill in field of study)* **context?**

If you did not miss a domain, then just click on "next".

You can suggest your own domain(s) below in the "Other domain" field.

Multiple answers possible – maximum 5

1. Other domain:

Etc.

Question 7 – Is there a topic that should be considered as a potential priority, but which you could not classify into the given domains earlier? You can also suggest overlapping topics that fit into more than one domain.

Please fill in only one topic per row

Multiple answers possible – Maximum 5 topics

Additional topic 1:

What is your particular interest in this topic?

What type of intervention / phenomena?

For whom? Who is the target group?

How should the success of the intervention be measured? Which factor needs to be assessed concerning this phenomenon?

Etc.

Section E: Selection of assessment criteria

Question 8 – Please indicate which criteria you believe are most important for assessing the importance of potential review topics in the field of … *(fill in field of study)***.**
Please first select the checkbox "Other" before proposing your own criterion (maximum three own proposed criteria)
You can hoover over a criterion to read its definition.

☐ Reduction of disease burden
☐ Health equity
☐ Lack of (good quality) evidence
☐ Effect on public health if successful
☐ Novelty of the concept
☐ Other 1:
☐ Other 2:
☐ Other 3:
☐ No answer

(These are just examples of assessment criteria and its definitions in the field of health. Please fill in own criteria above if you have better suitable ones)

When hoovering over the assessment criteria the following definitions will be shown:

1. Reduction of disease burden - The review topic will have a theoretical potential to reduce large portions of the existing disease burden.

2. Health equity - The review topic will likely reduce inequity in the accessibility of health interventions of health interventions

3. Effect on public health if successful – The review topic will have a substantial positive effect on public health research and/or practice.

4. Lack of (good quality) evidence – Synthesized evidence for the review topic is either not existing, lacking in quality, not up to date, or only applicable to a different context.

5. Novelty of the concept - The review topic represents a new or emerging area.

Section F: Experience with systematic reviews

You are almost finished.

In the following section we will ask you about your experience with systematic reviews. You do <u>not</u> need any knowledge or experience with systematic reviews in order to finish this survey.

The purpose of systematic reviews is to search, select and evaluate the best available research results for a particular topic – most often this is research about the effects of interventions or measures on the population or specific groups. In order to do so, the results of several studies are synthesized together.

<u>Question 9 – Have you ever been involved in systematic reviews?</u>
Multiple answers possible

- *No.*
- *Yes, I have worked as an author for a systematic review.*
- *Yes, I have worked as an advisor for a systematic review.*
- *Yes, I was a funder/contractor for a systematic review.*
- *No answer.*

<u>Question 10 – To what extent do you agree to the following statements?</u>

10a. I know what a systematic reviews is.

Disagree	Somehow disagree	Somehow agree	Agree

10b. I read the results of systematic reviews for my work.

Disagree	Somehow disagree	Somehow agree	Agree

10c. I read the results of systematic reviews from Cochrane for my work.

Disagree	Somehow disagree	Somehow agree	Agree

<u>Question 11 - To what extent do you agree with the following statements?</u>

Question 11 will only show up if the respondent provided a positive answer for question 10a.

	Disagree	Somehow disagree	Somehow agree	Agree
**11a. I believe that systematic reviews are important for** *... (fill in field of study)* **research.**				
**11b. I believe that systematic reviews are important for** *... (fill in field of study)* **practice.**				
**11c. The results of systematic reviews are helpful for my daily work**				
**11d. I know how to find relevant systematic reviews**				
**11e. I can interpret the results of systematic reviews**				
**11f. I find it difficult to understand the key message of systematic reviews**				
**11g. I have doubts about the results of systematic reviews**				
**17h. Systematic reviews question the independence in decision-making**				

Section G: Concluding question

Question 12 – Do you have any additional comments?

Many thanks for your important input in our research process.

Your answers will support us to gain more understanding about the research demands or preferences of stakeholders in ... *(fill in field of study)*. This will help us and other academia to guide future research.

Furthermore, we will use the results of this first online survey as input in the second survey of this Delphi study. You will receive an invitation for this second online survey ... *(fill in expected time for the start of the second online survey).*

For any further questions or remarks concerning the research project and/or the results once the study is finalised, please feel free to contact us:

… *(fill in contact details)*

You can now close of this browser!
