# Supplementary material for: Optimizing Research Impact: A Toolkit for Stakeholder‐Driven Prioritization of Systematic Review Topics
Source: Cochrane Evid Synth Methods. 2025 Aug 14;3(5):e70039. doi: 10.1002/cesm.70039 (PMC12362723; doi:10.1002/cesm.70039)
Supplement: Supplementary file 2 — Template online survey round 2. [file CESM-3-e70039-s002.pdf]

"Prioritising topics for systematic reviews"

Template for online survey round 2

(Fill in names and affiliations of the research team)

Section A: Introduction

Welcome to the second survey on: "Prioritising topics for systematic review"

Please read the following information before you agree on participating in this survey!

Thank you for your interest and participation in our second round of our Delphi study on prioritising topics for systematic reviews in the field … (*fill in field of study*).

In the first round of the Delphi study … *(fill in date)* you and many other stakeholders in … *(fill in geographical focus if applicable)* contributed to the creation of a list of topics which could be the foundation of systematic reviews in … (*fill in field of study*). Now … *(fill in inviting institutions)* invites you to the second Delphi round where you rate these topics and suggest their prioritisation.

This survey is conducted … *(fill in goal of the study).*

We would be delighted if you would also contribute to this second round. The time required is about 20 minutes.

In order to avoid multiple answers from individual participants, you can only fill out the survey once. However, on each of the following pages you can select the "continue later" option at the top of the website to save your answers to continue later. Your answers are saved for you even if you switch off your computer.

All data will be … *(fill in information regarding data protection and privacy)*.

If you have any questions or concerns, please do not hesitate to contact:

… *(fill in contact details)*

If you agree to this and would like to participate, then please click on "next".

Section H: General information – Round 2

Question 1 - Which position do you hold within your organisation?
Please choose the answer that best describes your situation in your main profession.

- *Employee*
- *Project leader*
- *Working group or department head*
- *Head of the organisation*
- *Other:*
- *No answer*

Question 2 – Which of the following options would you consider most appropriate for describing your profession?
Please choose the answer that best describes your situation in your main profession.

- *Research and/or higher education*
- *Governmental bodies and/or politics*
- *… (fill in field of study) related NGO & NPO and/or representatives of the public (for example: patient associations)*
- *Representative of … (fill in field of study) professionals*
- *… (fill in field of study) insurance*
- *Other:*
- *No answer*

Question 3 – At what level do you fulfil the majority of your activities?
Multiple answers possible

- *Local / Regional level*
- *National level*
- *International level*
- *No answer*

Question 4 – How many years have you been working in the field of … ***(fill in field of study)*****?**
If you do not consider yourself as active in this field of study, then please fill in "0"

Question 5 – Have you already participated in the first round of this Delphi study related to prioritising topics for systematic reviews?

- *Yes*
- *No*
- *I am not sure*
- *No answer*

Section J: Assess selected topics with selected criteria

Question 5 – Assessment of potential topics for systematic reviews based on stakeholder-refined criteria

Even if you did not participate in the first round, you can still participate in this survey.

The first round of the Delphi study took place in… *(fill in date)*. We have compiled and categorised all the proposals from the first round using content analysis. In total … *(fill in number of respondents in the first Delphi round)* stakeholders participated in the first round of the survey.

A table with all the results of our content analysis can be found here: … *(fill in weblink where the results of the content analysis for Delphi round 1 are presented).* The password is: … *(fill in password for accessing the link).*

In the next section, we ask you to rate the relevance of the listed topics in relation to a specific criterion. For example, an assessment criterion is … *(fill in subject related example).*

(If applicable)

In order to reduce the effort for you, you will only see a randomly generated selection of the topics and only rate those. In addition, we changed the order of the topics for the five criteria to minimize order distortion.

<u>Assessment criterion 1: *(… fill in desired criterion 1)*</u>

In the table below, you will find the topics for systematic reviews that you can assess with regard to the criterion … *(fill in criterion 1).*

For your information: The purpose of a systematic review is to summarize the best possible available research results on a specific issue, often on the effects of interventions or measures on the population or individual groups of people. For this purpose, the results of several studies are summarized.

Please indicate to what extent you agree with the following statement:

<u>"(… fill in desired operationalisation of criterion 1)"</u>

	Agree	Somehow agree	Somehow disagree	Disagree	I cannot assess this
Topic 1	○	○	○	○	○
Topic 2	○	○	○	○	○
Topic 3	○	○	○	○	○
Topic 4	○	○	○	○	○
..	○	○	○	○	○
..	○	○	○	○	○
..	○	○	○	○	○
..	○	○	○	○	○
Topic ...	○	○	○	○	○

<u>Assessment criterion 2: *(… fill in desired criterion 2)*</u>

In the table below, you will find the topics for systematic reviews that you can assess with regard to the criterion … *(fill in criterion 1).*

For your information: The purpose of a systematic review is to summarize the best possible available research results on a specific issue, often on the effects of interventions or measures on the population or individual groups of people. For this purpose, the results of several studies are summarized.

Please indicate to what extent you agree with the following statement:

<u>"(… fill in desired operationalisation of criterion 2)"</u>

	Agree	Somehow agree	Somehow disagree	Disagree	I cannot assess this
Topic 1	○	○	○	○	○
Topic 2	○	○	○	○	○
Topic 3	○	○	○	○	○
Topic 4	○	○	○	○	○
..	○	○	○	○	○
..	○	○	○	○	○
..	○	○	○	○	○
..	○	○	○	○	○
Topic ...	○	○	○	○	○

Assessment criterion 3: *(… fill in desired criterion 3)*

In the table below, you will find the topics for systematic reviews that you can assess with regard to the criterion … *(fill in criterion 1).*

For your information: The purpose of a systematic review is to summarize the best possible available research results on a specific issue, often on the effects of interventions or measures on the population or individual groups of people. For this purpose, the results of several studies are summarized.

Please indicate to what extent you agree with the following statement:

"(… fill in desired operationalisation of criterion 3)"

	Agree	Somehow agree	Somehow disagree	Disagree	I cannot assess this
Topic 1	○	○	○	○	○
Topic 2	○	○	○	○	○
Topic 3	○	○	○	○	○
Topic 4	○	○	○	○	○
..	○	○	○	○	○
..	○	○	○	○	○
..	○	○	○	○	○
..	○	○	○	○	○
Topic ...	○	○	○	○	○

<u>Assessment criterion 4: *(… fill in desired criterion 4)*</u>

In the table below, you will find the topics for systematic reviews that you can assess with regard to the criterion … *(fill in criterion 1).*

For your information: The purpose of a systematic review is to summarize the best possible available research results on a specific issue, often on the effects of interventions or measures on the population or individual groups of people. For this purpose, the results of several studies are summarized.

Please indicate to what extent you agree with the following statement:

<u>"(… fill in desired operationalisation of criterion 4)"</u>

	Agree	Somehow agree	Somehow disagree	Disagree	I cannot assess this
Topic 1	◯	◯	◯	◯	◯
Topic 2	◯	◯	◯	◯	◯
Topic 3	◯	◯	◯	◯	◯
Topic 4	◯	◯	◯	◯	◯
..	◯	◯	◯	◯	◯
..	◯	◯	◯	◯	◯
..	◯	◯	◯	◯	◯
..	◯	◯	◯	◯	◯
Topic ...	◯	◯	◯	◯	◯

Assessment criterion 5: ***(… fill in desired criterion 5)***

In the table below, you will find the topics for systematic reviews that you can assess with regard to the criterion … *(fill in criterion 1).*

For your information: The purpose of a systematic review is to summarize the best possible available research results on a specific issue, often on the effects of interventions or measures on the population or individual groups of people. For this purpose, the results of several studies are summarized.

Please indicate to what extent you agree with the following statement:

"(… fill in desired operationalisation of criterion 5)"

	Agree	Somehow agree	Somehow disagree	Disagree	I cannot assess this
Topic 1	◯	◯	◯	◯	◯
Topic 2	◯	◯	◯	◯	◯
Topic 3	◯	◯	◯	◯	◯
Topic 4	◯	◯	◯	◯	◯
..	◯	◯	◯	◯	◯
..	◯	◯	◯	◯	◯
..	◯	◯	◯	◯	◯
..	◯	◯	◯	◯	◯
Topic ...	◯	◯	◯	◯	◯

Section K: Weighting of the criteria

In the following section we are interested in the relative importance of the criteria you used in the previous section.

Question 6 – Please split 100 points into the following criteria.

We are interested in the relative importance of the criteria you used in the previous section. Now, we are asking you to distribute 100 points to the following criteria.
Please base your choice on how you rate the relative importance of the criteria in terms of assessing topics for systematic reviews in ... *(fill in field of study)*.
You can hover over a criterion to read its definition

Section L: Latest topics

At the beginning of the survey we gave you the link to the results of the first Delphi round. Some things concerning this field may have changed since the first survey. This may also influence the need for new topics for systematic reviews in this field.

Therefore, we want to give you the opportunity to suggest current topics which, in your opinion, should also be prioritized in systematic reviews.

Here you can find the link to the table with the aggregated results of the first Delphi round: … *(fill in weblink where the results of the content analysis for Delphi round 1 are presented).* The password is: … *(fill in password for accessing the link).*

Question 7 - Which potential topics should be considered as a high priority? Please do not hesitate to propose multiple topics.

The topics do not necessarily have to relate only to Switzerland. For each sector, the global context can also be considered.
Please fill in only one topic per row
Multiple answers possible – Maximum 5 topics

Topic 1:

What is your particular interest in this topic?

What type of intervention / phenomena?

For whom? Who is the target group?

How should the success of the intervention be measured? Which factor needs to be assessed concerning this phenomenon?

(Similar sub questions for topic two, three, four, and five)

Section M: Concluding question

Question 8 – Do you have any additional comments?

Many thanks for your important input in our research process.

Your answers will support us to gain more understanding about the research demands or preferences of stakeholders. This will help us and other academia to guide future research.

For any further questions or remarks concerning the research project and/or the results once the study is finalised, please feel free to contact us:

… *(fill in contact details)*

You can now close of this browser!
