# Supplementary material for: Optimizing Research Impact: A Toolkit for Stakeholder‐Driven Prioritization of Systematic Review Topics
Source: Cochrane Evid Synth Methods. 2025 Aug 14;3(5):e70039. doi: 10.1002/cesm.70039 (PMC12362723; doi:10.1002/cesm.70039)
Supplement: Supplementary file 5 — Modification guideline Online surveys priority setting for systematic review topics. [file CESM-3-e70039-s004.pdf]

Modification guideline for the online surveys on prioritising topics for systematic reviews

By Dyon Hoekstra

List of content

Introduction 1

Getting to know the survey structure 2

Tailoring the survey questions and structure 2

Activating the surveys to start the data collection 4

Additional tips 5

Introduction

The online surveys of our Delphi approach for prioritising topics for systematic reviews are easy-to-use and are developed in a way to allow the user to tailor it for their own research purposes. This modification guideline is developed specifically for our surveys. For a proper understanding it should be used in combination with our description in the article and in combination with the other supplementary material provided to the article.

Our surveys are available as part of our free toolkit as .lss files. The .lss files are exports of the entire surveys into a XML file (incl. the question groups, questions, subquestions, answers, conditions, and source codes) and are developed in the software LimeSurvey, however they can be easily imported and applied in different survey management software programs.

Overall, these survey management programs allow for structuring your surveys, design different question and answer types, and set conditions and rules that determine the logic of the survey.

Most open source survey management software programs provide a detailed manual for basic documentation and guidance on using the software effectively and is freely available online. These manuals, for example to design additional question or conditions in our survey, can be used additional to our modification guideline.

Although the provided survey structure files can be used in different program, this modification guideline uses terminology based on LimeSurvey, which can slightly differ from other survey management software. Below we explain the most important features of the survey structure and an explanation on how to make necessary adjustments to tailor the surveys for your own research purposes.

Getting to know the survey structure

To start with, after registering and login to your administrator account, you can use "Create survey" and then "**import**" to select our provided .lss file. This will upload and let you open the survey in order to tailor it accordingly. In general, it is advised to import the survey twice and to keep one original file untouched, as saved changes in the questions and conditions of the survey cannot always be undone.

Before starting to tailor the surveys, it is advised to skim through the different menus and use the "Preview survey" and/or "Preview question group" to get to know the survey structure better.

The main menu of the survey has two categories, Settings and Structure:

In the **settings** menu one can find and adjust general settings (such as language, administrator rights, and format of your surveys), text elements (such as the title and welcome page of your surveys), and other settings that can be adjusted related to data protection and participant privacy, and presentation styles. In order to make sure that the survey is functional as presented by us, it is advised to not make changes in the settings. Most importantly, leave the format setting to "group-by-group".

In the **structure** menu one will find the list of pre-programmed question groups and questions for our surveys. **Question groups**, sometimes consisting of multiple text elements and questions, help to logically structure and present a survey. Question groups can also be needed for adding certain conditions and controls the presentation (e.g. randomisation) of questions and allow for more complex survey routing based on previous answers. In our surveys it is for example relevant for the structure of the PICO questions. For each document, several questions are asked each representing a PICO element. In order to have a user-friendly survey that can analyse these elements as individual variables, it is advisable to program them as separate questions. However, for a logical and respondent-friendly presentation of the questions, these separate questions are much better presented together, using conditions for answering them element-by-element.

Tailoring the survey questions and structure

In order to tailor all the text elements and questions in the survey to your specific research focus, you just have to fill in the open spots we have created in the text elements and questions. For example, the field of the study and the contact details of the study / survey coordinator needs to be filled in and later in the survey answer options based on your research focus needs to be adjusted.

Use the "Edit" button to change a piece text. We advise to fill in or adjust the text only in the **source code**. After pressing "Edit" one can find the toolbar of the question text window. The second icon from the left will bring you to the source code of the text. In the source code, general formatting rules are programmed, but also more detailed visualisation and logic rules

are occasionally programmed in the source code, for a smooth functioning of the survey. In order to not lose the pre-programmed formatting and logic one is advised to make textual changes in the source code.

After each change in the survey one needs to **save** it directly (top right corner of the screen) as changes are not automatically saved when leaving a page.

If you open a single question for editing, you can find in the right column information about the question name, question type, and setting like if the question is mandatory or not if it holds certain conditions. This information and these settings are also already pre-programmed and do not need to be changed.

Depending on the question type, one will find “Subquestions” and/or “Answer options” below the question´s text:

Answer options (or response options) refer to the choices or selections provided to respondents for a particular question in a survey. Answer options are used to gather specific responses or preferences from survey respondents. For example, in question one of our survey, the answer options represent the different occupational positions out of which the respondent can choose one.

The **subquestions** are a way to create more complex and detailed survey questions by breaking them down into smaller, related components. Subquestions are associated with the main (or parent) question and are used to gather specific details or information related to the main question. With some question types such as multiple-choice questions (see question 3 in our first Delphi survey), they simply represent the response options. With other question types such as array questions, they represent the statement that needs to be assessed under the main question. The answer options for an array question then represents the “value” of the scale items on which the statements are assessed. For example, in question 10 of our first Delphi round survey: The main question (array type) is “Question 10 – To what extent do you agree to the following statements?”. The subquestions are the statements, such as “10a. I know what a systematic review is.” And the answer options name the values of the 4-point Likert scale items (Disagree; Somehow disagree; Somehow agree; Agree).

In some cases, a code text is inserted in the subquestion to allow for a definition to pop-up for the respondents when they hoover over the option. For example, in question 8, the assessment criteria that can be selected are listed as subquestions. The first part of the following code is the text that pops-up when hoovering over the option, the last part of the code is the title of the option that can be selected (Code: Reduction of disease burden)

In the overview of some questions one will see a highlighted “**Condition**”. Conditions are rules that help to determine the logic of the survey. e.g. when jumping to specific questions based on responses and **relevance equations** that make questions only relevant if a specific previous

answer is given. The text fields for the relevance equations can be found in the column next to the subquestions.

Example 1: In question 5 of our first Delphi survey, respondents are asked to propose topics they believe are important for the priority setting. Once, and only if, the text field for topic 1 is filled in, additional questions pop-up to identify the different element of the PICO.

Example 2: An important part of our Delphi surveys is the rating of topics in the second Delphi round by assessment criteria (Section J in the survey structure). In case there are many topics from the first Delphi round, we strongly recommend to limit the number and randomise the topics that each respondent needs to assess. In order to so, we added a so called "**equation question**" in each question group of section J (named "J1rand …"). Rand() generates a random integer. In our example "rand (0,5)" is used, which means that a random number is created between 1 and 5 each time (on average a 1 out 5 chance a certain number is created). This randomly triggered number is connected to the relevance equation at the subquestions, which can be found in the same question group in the question that is above the equation question, in our survey called "RatingCrit…". The code at the relevance equation indicates, that if the randomly generated number is 1, then the subquestion is presented to the respondent and needs to be answered. Hence, on average 1 in 5 subquestions (in our example topics) need to be assessed, leaving it random fate which ones these will be. The 0,5 in the equation question can be adjusted if you want to change the likelihood a subquestion/topic needs to be selected.

Activating the surveys to start the data collection

After everything is programmed and set, the respondents need to be invited to fill in the survey. In order to ease this process, one can use the survey management software to invite (and if applicable) remind the respondents automatically:

The contact details of the respondents need to be filled in the "survey participants" menu under "settings". One can choose to use a closed-access mode or an open-access code. The closed-access option creates a participant table, with all the relevant contact information of the respondents, which allows you to send invitations and follow the progress (still anonymously) through the software itself and only allows respondents who provide an access code to participate in the survey.

Thereafter, the email template(s) for the invitation(s) and reminder(s) need(s) to be inserted in the "Email templates" menu. In the template, the name of the respondent, the survey title, the name(s) of the study coordinator(s) and the weblink to the survey, can be automatically generated. This template is already filled in for the provided surveys, it just needs to be updated at the settings of the surveys to be presented properly. Also, an automatically generated token for accessing the survey anonymously can be added in the template. This token can be used to send reminders only to the participants who didn´t fill in the survey yet in an anonymous way. Furthermore, it can allow the respondents to save their progress in the survey and continue another time, if preferred.

After the survey, the participant table, and the email templates have been finalised, the survey can be activated and invitations can be (automatically or manually) send to the respondents.

Additional tips

- As undoing changes that have been applied is problematic in most survey management programs, one can export original questions or questions groups from an old version of the survey to import them into the newly adjusted survey again. In this way, only changes to that specific question or question group are lost and one can start to tailor the specific question again.
- When questions, question groups, subquestions, or answer options are integrated while tailoring the surveys, the used survey management software might change question or answer codes. This means that these codes also have to be adjusted in the conditions and relevance equations, otherwise they are not functioning.
- If one wants to use multiple versions of the survey, for example different languages, then this needs to be first set in the “general settings” menu. After the languages are selected, the survey management software will automatically copy the survey structure and questions for each language. The user of course will need to translate all the text elements, questions, subquestions, and answer options. As this might change the question and answer codes as well, one needs to ensure the conditions are updated accordingly. One should see the different versions as separate surveys (with each another access link for respondents as well), which means changes in the one will not automatically change the other version. Therefore, it is advisable to only add the other versions at the end after everything is programmed, hence (if necessary) only the text elements are left for modification.
- In order to insert a lot of response options (either as subquestions or as answer options) one can use the function “Quick add” to copy all the options from a different file and insert them all at once, instead of manually inserting each one of them individually. This is for example relevant to the rating question (Section J in the second survey) in which likely a long list of topics that need to be rated, have to be filled in.
- If you have specific customization needs or advanced requirements, most commonly used survey management programs provide detailed manuals and Q & A sections to solve any question one might have. In LimeSurvey for example, one can use the Expression Manager to achieve some level of customization without directly editing the source code.
